# Patterns of Internet Use, Loneliness, and Mental and Physical Health Among Korean Baby Boomers

**DOI:** 10.1093/geroni/igaa057.1011

**Published:** 2020-12-16

**Authors:** Jeehoon Kim, Jwakyum Kim, Ji Hyun Lee, Gyounghae Han

**Affiliations:** 1 Idaho State University, Pocatello, Idaho, United States; 2 Kkottongnae University, Cheongju, Republic of Korea; 3 Florida State University College of Medicine, Tallahassee, Florida, United States; 4 Seoul National University, Seoul, Republic of Korea

## Abstract

Introduction. Korean Baby Boomers are more affluent and gained higher education than past generations. Their relatively younger age (born between 1955 and 1963), coupled with more personal resources can help the Boomers have easier access to digital devices, but little is known about their patterns of online activities and how diversity in online activities is associated with health status among Korean Boomers. Methods. Using data from the 2012 Korean Baby Boomers Panel Study (n=3,272), we conducted latent class analysis based on ten online activities. Three classes were identified with different patterns of online activities; namely, maximizers (n=315), selective users (n=1,435), and rare users (n=1,522). Multinomial logistic regression analysis was performed to examine factors associated with different groups. Ordered probit regression and multiple regression analysis were employed to predict depressive symptoms and self-reported health of Korean Boomers, respectively. Results. Selective users used the internet for information search, e-mail, SNS, streaming music, or gaming, whereas maximizers engaged actively on all activities. Rare users were minimally engaged in information search. Both selective users and maximizers were more actively engaged with social relationships and social group activities compared to rare users. Maximizers were more likely to experience loneliness than rare users. Selective users were less likely to report depressive symptoms and had better health than rare users. Discussion. Moderate engagement with digital technology supported Korean Boomers in maintaining social relationships and better health. Intervention strategies for alleviating loneliness and preventing social isolation for maximizers, thus helping them maintain better health, will be discussed.

